# A Simple Approach for Monitoring Business Service Time Variation

**DOI:** 10.1155/2014/238719

**Published:** 2014-05-07

**Authors:** Su-Fen Yang, Barry C. Arnold

**Affiliations:** ^1^Department of Statistics, National Chengchi University, Taipei 116, Taiwan; ^2^Department of Statistics, University of California, Riverside, CA 92521, USA

## Abstract

Control charts are effective tools for signal detection in both manufacturing processes and service processes. Much of the data in service industries comes from processes having nonnormal or unknown distributions. The commonly used Shewhart variable control charts, which depend heavily on the normality assumption, are not appropriately used here. In this paper, we propose a new asymmetric EWMA variance chart (EWMA-AV chart) and an asymmetric EWMA mean chart (EWMA-AM chart) based on two simple statistics to monitor process variance and mean shifts simultaneously. Further, we explore the sampling properties of the new monitoring statistics and calculate the average run lengths when using both the EWMA-AV chart and the EWMA-AM chart. The performance of the EWMA-AV and EWMA-AM charts and that of some existing variance and mean charts are compared. A numerical example involving nonnormal service times from the service system of a bank branch in Taiwan is used to illustrate the applications of the EWMA-AV and EWMA-AM charts and to compare them with the existing variance (or standard deviation) and mean charts. The proposed EWMA-AV chart and EWMA-AM charts show superior detection performance compared to the existing variance and mean charts. The EWMA-AV chart and EWMA-AM chart are thus recommended.

## 1. Introduction

Control charts are commonly used tools in process signal detection to improve the quality of manufacturing processes and service processes. In the past few years, more and more statistical process control techniques have been applied to the service industry, and control charts are also becoming an effective tool in improving service quality. There have been a few studies in this area, like those of MacCarthy and Wasusri [1], Tsung et al. [2], and Ning et al. [3]. Much service process data come from processes with variables having nonnormal or unknown distributions so the commonly used Shewhart variables control charts, which depend on a normality assumption, are not suitable. Hence the following question arises: “how to monitor the process with nonnormal or unknown distribution data?” Some research has been done to deal with such a situation; see, for example, Ferrell [4]; Bakir and Reynolds [5]; Amin et al. [6]; Chakraborti et al. [7]; Altukife [8, 9]; Bakir [10]; Chakraborti and Eryilmaz [11]; Chakraborti and Graham [12]; Chakraborti and van der Wiel [13]; Li et al. [14]; and Zou and Tsung [15]. Little research has been done to deal with process variability monitoring; see, for example, Das and Bhattacharya [16].

A major drawback of the previous nonparametric approaches is that they are not easy for practitioners to apply because they are not statisticians and do not quite understand the proper way to implement the schemes. Yang et al. [17] proposed a new sign chart for variables data to monitor the deviation of the process measurement from the target without the assumption of a normal process distribution or a distribution of known form. Yang and Cheng [18] proposed a CUSUM mean chart to monitor small shifts in the process mean. Yang et al. [19] addressed a new mean chart based on a simple statistic to monitor the shifts of the process mean. Their approaches are quite easy to use, and even easier than some of the above published nonparametric approaches. However, the mean charts based on an asymmetric binomial distribution (i.e., when *p* ≠ 1/2)do not exhibit a regular in-control run length. Moreover the corresponding out-of-control average run lengths do not uniformly decrease as sample size increases as they should. Further, Yang and Cheng [18], Yang et al. [17], and Yang et al. [19] did not consider a variance chart.

In this paper, we propose an improved asymmetric EWMA mean chart (EWMA-AM chart) and a new asymmetric EWMA variance chart (EWMA-AV chart) for variables data to effectively monitor the process mean and variance simultaneously. The approach is still quite easy to use and has better detection ability than the existing mean and standard deviation charts. The paper is organized as follows. In Section 2, we propose the exponentially weighted moving average (EWMA) chart, EWMA-AM chart, to detect the out-of-control process mean and measure its performance. In Section 3, we discuss the construction of a newly proposed EWMA-AV chart to detect the out-of-control process variance and its performance. In Section 4, we propose to combine the two EWMA charts, EWMA-AM chart and EWMA-AV chart, to detect both the out-of-control mean and variance and measure the performance. In Section 5, we describe the estimates for the unknown process mean and variance. In Section 6, a numerical example of a service system in a bank branch was used to construct the proposed EWMA-AM chart and EWMA-AV chart to monitor the quality of service time and their performance compared with those of existing charts.  Section 7 summarizes the findings and provides a recommendation.

## 2. The Proposed EWMA-AM Chart

Assume that a critical quality characteristic, *X*, has a mean *μ* and variance *σ* ^2^.

Following Yang et al. [17], let *Y* = *X* − *μ* and *p* = *P*(*Y* > 0) = the “Process Proportion.” If the process was in control then *p* = *p*_*m*0_, and if the process was out of control, that is, *μ* had shifted; then *p* = *p*_*m*1_ ≠ *p*_*m*0_. If *p*_*m*0_ is not given, it will be estimated using a preliminary data set.

To monitor the process mean, a random sample of size *n*_1_, *X*_1_, *X*_2_,…, *X*_*n*_1__, is taken from *X*. Define

(1) $$\begin{array}{c} Y_{j}=X_{j}-\mu ,\;I_{j}=\{\begin{array}{cc} 1, & \text{if}\;Y_{j}>0,\\ 0, & \text{otherwise}, \end{array}\;j=1,2,\ldots ,n_{1}. \end{array}$$

Let *M*_*t*_ be the total number of *Y*_*j*_> 0 at time *t*; then *M*_*t*_ = ∑_*j*=1_^*n*_1_^ *I*_*j*_ would follow a binomial distribution with parameters (*n*_1_, *p*_*m*0_) for an in-control process.

Based on the distribution of *M*, the *M* chart could be constructed to monitor the process proportion. Monitoring the process mean shifts is equivalent to monitoring the changes in process proportion.

### 2.1. The Control Limits of EWMA-AM Chart

To overcome the defects of using the symmetric mean chart and to have superior performance in detecting small shifts in the process mean, a new improved asymmetric EWMA mean chart (EWMA-AM chart) is proposed. The EWMA control charts have been demonstrated to have better performance for detecting small shifts in process parameters when compared with Shewhart-type charts (e.g., see [20]). The new improved asymmetric EWMA mean chart (EWMA-AM chart) has upper control limit (UCL_EWMA_*M*__), central line (CL_EWMA_*M*__), and lower control limit (LCL_EWMA_*M*__) defined as follows:

(2) $$\begin{array}{c} {\text{UCL}}_{{\text{EWMA}}_{M}}=n_{1}p_{m0}+k_{3}\sqrt{\frac{\lambda_{1}}{\left(2-\lambda_{1}\right)}n_{1}p_{m0}\left(1-p_{m0}\right)},\\ {\text{CL}}_{{\text{EWMA}}_{M}}=n_{1}p_{m0},\\ {\text{LCL}}_{{\text{EWMA}}_{M}}=n_{1}p_{m0}-k_{4}\sqrt{\frac{\lambda_{1}}{\left(2-\lambda_{1}\right)}n_{1}p_{m0}\left(1-p_{m0}\right)}, \end{array}$$

where *k*_3_ and *k*_4_ are appropriately chosen coefficients for the UCL_EWMA_*M*___  _and LCL_EWMA_*M*__, respectively.

If any monitoring statistic EWMA_*M*_*t*__ exceeds UCL_EWMA_*M*__ or if EWMA_*M*_*t*__ falls below LCL_EWMA_*M*__, the process is deemed to exhibit some out-of-mean-control signal.

The monitoring statistic EWMA_*M*_*t*__ based on the statistic *M* is defined as follows:

(3) EWMAMt=λMt+(1−λ1)EWMAMt−10<λ1≤1, t=1,2,3,….

Let the starting value, EWMA_*M*_0__, be the mean of *M*; that is, EWMA_*M*_0__ = *n*_1_ *p*_*m*0_ for an in-control process. Hence the mean and variance of EWMA_*M*_*t*__ are

(4) $$\begin{array}{c} E\left({\text{EWMA}}_{M_{t}}\right)=n_{1}p_{m0},\\ \mathrm{Var}\left({\text{EWMA}}_{M_{t}}\right)=\frac{\lambda_{1}\left[1-{\left(1-\lambda_{1}\right)}^{2t}\right]}{2-\lambda_{1}}\left(n_{1}p_{m0}\left(1-p_{m0}\right)\right). \end{array}$$

The asymptotic variance of EWMA_*M*_*t*__ is

(5) $$\begin{array}{c} \mathrm{Var}\left(\text{EWM}{\text{A}}_{M_{t}}\right)=\frac{\lambda_{1}}{2-\lambda_{1}}\left(n_{1}p_{m0}\left(1-p_{m0}\right)\right). \end{array}$$

To measure the performance of the proposed new EWMA-AM chart, we calculated the average run length (ARL). The in-control ARL, ARL_*m*0_, of the EWMA-AM chart depends on the values of *n*_1_, *k*_3_, *k*_4_, *λ*_1_, and *p*_*m*0_. The two parameters, *k*_3_ and *k*_4_, for a specified *λ* and *n*_1_ = 10(1)30 are chosen to satisfy a required in-control average run length (ARL_*m*0_) ≈ 370 using a Markov chain approach [21], and the calculation procedure follows that used in Yang [22].  Table 1 shows the values of *k*_3_ and *k*_4_ with *λ*_1_ = 0.05 and ARL_*m*0_ ≈ 370 for *n*_1_ = 10(1)30 and *p*_*m*0_ = 0.1(0.1)0.9.

### 2.2. The Out-of-Control Average Run Lengths of the EWMA-AM Chart

For the out-of-control process it is assumed that the mean *μ* has shifted, and the process proportion has become *p*_*m*1_ (≠*p*_*m*0_). Let ARL_*m*1_ be the out-of-control ARL of the EWMA-AM chart. A small ARL_*m*1_ indicates superior out-of-control detection performance of the control chart. Similar to the calculation of ARL_*m*0_, ARL_*m*1_ can be obtained by a Markov chain approach for various *p*_*m*1_ under a specified *p*_*m*0_, *n*_1_, *k*_3_, *k*_4_, and *λ*_1_.

The ARL_*m*1_s of the EWMA-AM chart under the combinations of *n*_1_ = 10(1)30, *p*_*m*1_ = 0.1(0.1)0.9 with ARL_*m*0_ ≈ 370 and *λ*_1_ = 0.05 when *p*_*m*0_ = 0.1, 0.3, 0.5 and the corresponding *k*_3_ and *k*_4_ are calculated and listed in Tables 2, 3, and 4, respectively.

It can be seen that the values of the ARL_*m*1_s behave appropriately; that is, they change inversely with *n*_1_, and the ARL_*m*1_s decrease when *p*_*m*1_ is far away from *p*_*m*0_.

When one compares the out-of-control average run lengths between the EWMA-AM chart (Table 4) and the symmetric EWMA mean chart (Table 5) in Yang et al. [17], for example, with *λ*_1_ = 0.05, *p*_*m*0_ = 0.5, and ARL_*m*0_ ≈ 370, one finds that the EWMA-AM chart shows slightly superior out-of-control detection performance.

## 3. The Proposed EWMA-AV Chart

To monitor the process variance, a random sample of size *n*_2_, *X*_1_, *X*_2_,…, *X*_*n*_2__, is taken from the process, *X*. Assume that the sample size *n*_2_ is even for convenience (if not, delete one observation).

Define

(6)  Y1∗=(X2−X1)22, Y2∗=(X4−X3)22,   ⋮Yn2/2∗=(Xn2−Xn2−1)22,E(Yj′∗)=σ2, j′=1,2,…,0.5n2,Ij′={1,if  Yj′∗>σ20,otherwise for  j′=1,2,…,0.5n2.

Let *V* be the total number of *Y*_*j*′_* > *σ* ^2^; then *V* = ∑_*j*′=1_^0.5*n*_2_^ *I*_*j*′_ will have a binomial distribution with parameters (0.5*n*_2_, *p*_*v*0_) for an in-control process where *p*_*vo*_ = *P*(*Y*_*j*′_* > *σ* ^2^). The value of *p*_*v*0_ will depend on the distribution of the *X*_*i*_'s. For example, if the *X*_*i*_'s are normally distributed then *p*_*vo*_ = *P*(*Y*_*j*′_* > *σ* ^2^) = *P*(*Z* ^2^ > 1) where *Z* ~ *N*(0,1). Thus in this case *p*_*v*0_ = 0.3147. If the distribution of *X*_*n*_2__–*X*_*n*_2_−1_ is unimodal, as it frequently is, the version of the Tchebychev inequality for unimodal variables implies that the quantity *p*_*v*0_ is bounded above by 4/9. The value of *p*_*v*0_ can be arbitrarily small but it usually will be in the range 0.25–0.50. Note that, although the resulting chart is a 0.5*n*_2_ *p*_*v*0_ chart, this is a new chart in that the binomial variable is not the count of nonconforming units in the sample but rather the number of pairs of *X* values in a sample that is in control with respect to the process variance. Monitoring process variance shifts are equivalent to monitoring the changes in process proportion, *p*_*v*0_. For the in-control process, we may define the monitoring statistic *V*_*t*_ as the number of *Y*_j_*'s>*σ* ^2^ at time *t*; hence, *V*_*t*_ ~ *B*(0.5*n*_2_, *p*_*v*0_).

### 3.1. The Control Limits of EWMA-AV Chart

The variance chart based on an asymmetric binomial distribution has similar defects to those of the corresponding mean chart. It exhibits an irregular in-control average run length and its out-of-control average run length does not change appropriately as sample size increases. A new EWMA asymmetric variance chart (EWMA-AV chart) based on the statistic *V* is constructed as follows:

(7) $$\begin{array}{c} \text{UC}{\text{L}}_{{\text{EWMA}}_{V}}=0.5n_{2}p_{v0}+L_{3}\sqrt{\frac{\lambda_{2}}{\left(2-\lambda_{2}\right)}0.5n_{2}p_{v0}\left(1-p_{v0}\right)},\\ \text{C}{\text{L}}_{{{\text{EWMA}}_{V}}_{\;}}=0.5n_{2}p_{0v},\\ \text{LC}{\text{L}}_{{\text{EWMA}}_{V}}=0.5n_{2}p_{v0}-L_{4}\sqrt{\frac{\lambda_{2}}{\left(2-\lambda_{2}\right)}0.5n_{2}p_{v0}\left(1-p_{v0}\right)}, \end{array}$$

where *L*_3_ and *L*_4_ are appropriately chosen coefficients for UCL_EWMA_*V*__ and LCL_EWMA_*V*__.

If any monitoring statistic EWMA_*V*_*t*__ exceeds UCL_EWMA_*V*__ or if EWMA_*V*_*t*__ < LCL_EWMA_*V*__, the process is deemed to show some out-of-variance-control signals.

The monitoring statistic EWMA_*V*_*t*__ based on the statistic *V*_*t*_ is thus defined as follows:

(8) EWMAVt=λ2Vt+(1−λ2)EWMAVt−10<λ2≤1, t=1,2,….

Let the starting value, EWMA_*V*_0__, be the mean of *V*; that is EWMA_*V*_0__ = *n*_2_ *p*_*v*0_ for an in-control process. Hence the mean and variance of EWMA_*V*_*t*__ are

(9) $$\begin{array}{c} E\left(\text{EWM}{\text{A}}_{V_{t}}\right)=0.5n_{2}p_{v0},\\ \mathrm{Var}\left(\text{EWM}{\text{A}}_{V_{t}}\right)=\frac{\lambda_{2}\left[1-{\left(1-\lambda_{2}\right)}^{2t}\right]}{2-\lambda_{2}}\left(0.5n_{2}p_{v0}\left(1-p_{v0}\right)\right). \end{array}$$

The asymptotic variance of EWMA_*V*_*t*__ is

(10) $$\begin{array}{c} \mathrm{Var}\left(\text{EWM}{\text{A}}_{V_{t}}\right)=\frac{\lambda_{2}}{2-\lambda_{2}}\left(0.5n_{2}p_{v0}\left(1-p_{v0}\right)\right). \end{array}$$

The ARL is also used to measure the performance of the proposed new EWMA-AV chart. The in-control ARL, ARL_*v*0_, of the EWMA-AV chart depends on the values of *n*_2_, *L*_3_, *L*_4_, *λ*_2_, and *p*_*v*0_. In a manner similar to that used to obtain *k*_3_ and *k*_4_ in Section 2, the two parameters, *L*_3_ and *L*_4_, may be obtained when the values of 0.5*n*_2_ and *p*_*v*0_ are given and hence one sets ARL_*v*0_ ≈ 370 using Markov chain approach. The *L*_3_ and *L*_4_ with *λ*_2_ = 0.05 and ARL_*v*0_ ≈ 370 for 0.5*n*_2_ = 5(1)15 and *p*_*v*0_ = 0.1(0.1)0.4 are illustrated in Table 6.

### 3.2. The Out-of-Control Average Run Lengths of the EWMA-AV Chart

To measure the out-of-control detection performance of the EWMA-AV chart, we calculate the out-of-control average run length (ARL_*v*1_) for the combinations of 0.5*n*_2_ = 5(1)15, out-of-control proportion *p*_*v*1_ = 0.1(0.1)0.9 with ARL_*v*0_ ≈ 370 and *λ*_2_ = 0.05 for adopting *p*_*v*0_ = 0.1, 0.4, and corresponding *L*_3_ and *L*_4_ using Markov chain approach. The results are listed in Tables 7 and 8.

The results look reasonable since the values of the ARL_*v*1_s change inversely with *n*_2_, and the ARL_*v*1_s decrease when *p*_*v*1_ is far away from *p*_*v*0_.

## 4. Performance Measurement of Using EWMA-AM Chart and EWMA-AV Chart Simultaneously

Using both the EWMA-AM chart and EWMA-AV chart we may monitor the process mean and variance simultaneously. We will use the overall average run length (ARL) to measure the performance of using both the EWMA-AM chart and EWMA-AV chart.

Since the statistics EWMA_*M*_ and EWMA_*V*_ are independent, the in-control overall ARL, ARL_0_, of the newly proposed EWMA-AM and EWMA-AV charts is well approximated as follows (see Hawkins (1992)):

(11) ARL0 =11/ARLm0+1/ARLv0−((1/ARLm0)(1/ARLv0)) ≈11/ARLm0+1/ARLv0.

The ARL_0_ of using both the EWMA-AM chart and EWMA-AV chart with any combinations of (*n*_1_, *n*_2_) and (*p*_*m*0_, *p*_*v*0_) are all approximately 185 because of

(12) $$\begin{array}{c} \text{AR}{\text{L}}_{0}\approx \frac{1}{1/\text{AR}{\text{L}}_{m0}+1/\text{AR}{\text{L}}_{v0}}=\frac{1}{1/370+1/370}. \end{array}$$

If the process is out-of-control because the mean *μ* has shifted, the process proportion becomes *p*_*m*1_ (≠*p*_*m*0_). If the process is out-of-control because the variance *σ* ^2^ has changed, the process proportion becomes *p*_*v*1_(≠*p*_*v*0_).

The out-of-control ARL, ARL_1_, when using the EWMA-AM chart and EWMA-AV chart simultaneously can be calculated approximately using

(13) $$\begin{array}{c} \text{AR}{\text{L}}_{1}\approx \frac{1}{1/\text{AR}{\text{L}}_{m1}+1/\text{AR}{\text{L}}_{v1}}. \end{array}$$

We calculate approximate ARL_1_s for the combinations of *n*_1_ = 10(2)30, 0.5*n*_2_ = 5(1)15, *p*_*m*1_ = 0.1, 0.3, 0.7, and *p*_*v*1_ = 0.1, 0.3, 0.7 with *p*_*m*0_ = 0.1, *p*_*v*0_ = 0.1, *λ*_1_ = *λ*_2_ = 0.05, and ARL_0_ ≈ 185. The results are listed in Table 9. The ARL_1_s for the combinations of *n*_1_ = 10(2)30, 0.5*n*_2_ = 5(1)15, *p*_*m*1_ = 0.1, 0.3, 0.7, and *p*_*v*1_ = 0.1, 0.3, 0.7 with *p*_*m*0_ = 0.3, *p*_*v*0_ = 0.1, ARL_0_ ≈ 185, and *λ*_1_ = *λ*_2_ = 0.05 are calculated and listed in Table 10. The ARL_1_s for the combinations of *n*_1_ = 10(2)30, 0.5*n*_2_ = 5(1)15, *p*_*m*1_ = 0.1, 0.3, 0.7, and *p*_*v*1_ = 0.1, 0.3, 0.7 with *p*_*m*0_ = 0.5, *p*_*v*0_ = 0.4, ARL_0_ ≈ 185, and *λ*_1_ = *λ*_2_ = 0.05 are calculated and listed in Table 11. In Tables 9–11, we observe that the ARL_1_ changes inversely with *n*_1_ and *n*_2_, and the ARL_1_ decreases when *p*_*m*1_ is far away from *p*_*m*0_ and/or *p*_*v*1_ is far away from *p*_*v*0_. This contrasts with the previously noted inappropriate behavior exhibited by the *V*and the *M* charts.

## 5. When Population Mean and Variance Are Unknown

When the in-control process mean, *μ*, and the process variance, *σ* ^2^, are unknown, and hence the in-control process proportions, *p*_*m*0_ and *p*_*v*0_, are unknown, we can use the following two preliminary independent sample data sets:

(14) $$\begin{array}{c} X_{t1},X_{t2},\ldots ,X_{tn_{1}},\;t=1,2,\ldots ,k,\\ X_{tn_{1}+1},X_{tn_{1}+2},\ldots ,X_{tn_{1}+n_{2}},\;t=1,2,\ldots ,k \end{array}$$

from *k* sampling periods, each with an even number of observations, *n*_1_ and *n*_2_, to estimate them (see, e.g., [20]); that is

(15) μ^=x¯¯=∑t=1k∑j=1n1xtjkn1,  σ^=S¯c4=∑t=1kStc4k,

where

(16) p^m0=∑t=1k(Mt/n1)k,  p^v0=∑t=1k(Vt/0.5n2)k,St=∑j=1n2(Xtj−Xt¯)2n2−1t=1,…,k,c4=(2n2−1)0.5Γ(0.5n2)Γ(0.5(n2−1)),  S¯=∑t=1kStk.

The EWMA-AVand EWMA-AM charts are thus constructed using these estimated values of *p*_*m*0_ and *p*_*v*0_. The statistics EWMA_*M*_ and EWMA_*V*_ corresponding to the samples of sizes *n*_1_ and *n*_2_ are plotted on the resulting EWMA-AV and EWMA-AM charts simultaneously. If no points fall outside their control limits, then we would deem the process to be in control.

## 6. Example

We will use an example from Yang et al. [17] to illustrate the new EWMA-AV and EWMA-AM charts.

Service time is an important quality characteristic in the banking industry. To measure the efficiency in the service system of a bank branch, the in-control sampling service times (unit: minutes) are measured from twenty counters every day for 15 days. That is, fifteen samples of size *n*_1_ + *n*_2_ = 20, where *n*_1_ = *n*_2_ = 10, are available. This in-control data has been analyzed assuming a nonnormal distribution. For each sample, the first ten observations illustrated in Table 12(a) are used to calculate the EWMA_*M*_ statistic and the last ten observations illustrated in Table 12(b) are used to calculate the EWMA_*V*_ statistic.

To construct the EWMA-AV and EWMA-AM charts, the variance and mean of the service time are estimated by ${\left(\bar{S}/c_{4}\right)}^{2}$ and $\bar{\bar{x}}$ using the fifteen samples in Tables 12(b) and 12(a), respectively. The estimate of the variance is σ2^=(S¯/c4)2=30.159 and the estimate of the mean is μ^=x¯¯=5.77. For each sample in Table 12(b), the monitoring statistic EWMA_*V*__  _ = total number of (*Y*_j_* > 30.159), *j* = 1,2,…, 10, is calculated. For each sample in Table 12(a), the monitoring statistic EWMA_*M*_ = total number of (*Y*_*j*_ > 5.77), *j* = 1,2,…, 10, is calculated. Hence, the estimates of proportions (*p*_*m*0_, *p*_*v*0_) are (${\hat{p}}_{m0}=\left(\sum_{t=1}^{15}M_{t}/10\right)/15=0.39$, ${\hat{p}}_{v0}=\left(\sum_{t=1}^{15}V_{t}/5\right)/15=0.24$). The EWMA-AV and EWMA-AM charts with *λ*_1_ = *λ*_2_ = 0.05 are constructed as follows based on the fifteen in-control samples, respectively.

 The EWMA-AV chart:
  (17) $$\begin{array}{c} \text{UC}{\text{L}}_{\text{EWMA-}V}=1.59,\;\;\text{LC}{\text{L}}_{\text{EWMA-}V}=0.83. \end{array}$$
 The EWMA-AM chart:
  (18) $$\begin{array}{c} \text{UC}{\text{L}}_{\text{EWMA-}M}=4.51,\;\;\text{LC}{\text{L}}_{\text{EWMA-}M}=3.28. \end{array}$$

**Figure 1:**
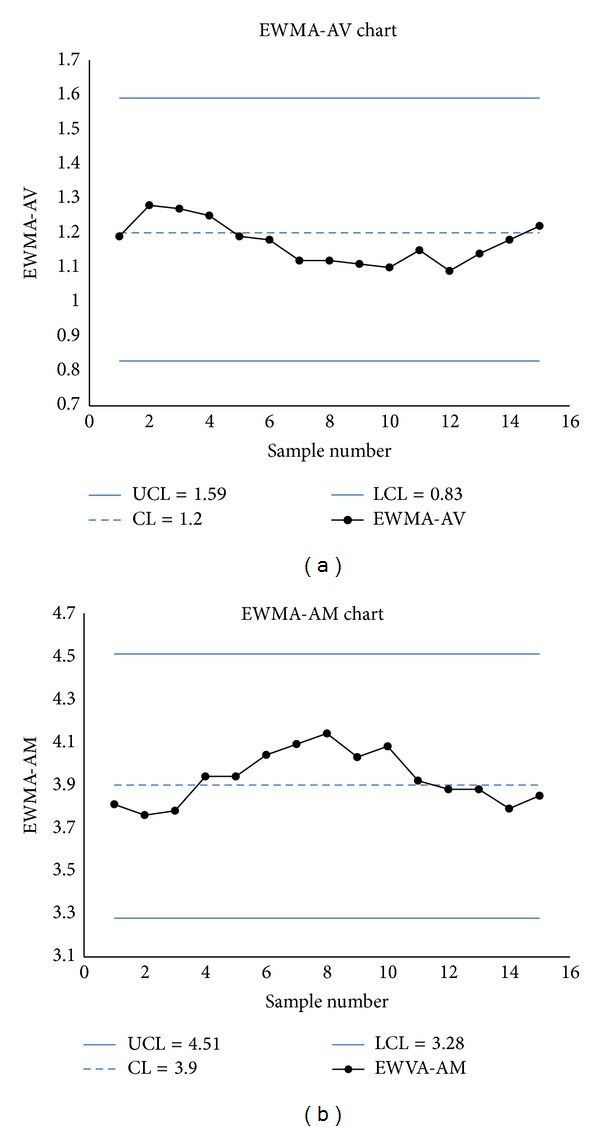
(a) The EWMA-AV chart. (b) The EWMA-AM chart.

The monitoring statistics EWMA_*V*_ and EWMA_*M*_ are calculated (see Tables 12(b) and 12(a)). The EWMA-AV and EWMA-AM charts show no signals (see Figures 1(a) and 1(b)).

For comparison, we constructed the corresponding Shewhart mean and standard deviation ($S\text{-}\bar{X}$) charts and the transformed $S\text{-}\bar{X}$ charts by applying *X* ^0.278^ transformation because *X* is a right-skewed distribution (see [20]). The Shewhart $S\text{-}\bar{X}$ charts, the transformed $S\text{-}\bar{X}$ charts, and the EWMA-*S* and EWMA-$\bar{X}$ charts are constructed with bounds as follows:

(19) $$\begin{array}{c} \text{UC}{\text{L}}_{S}=8.80,\;\;\text{LC}{\text{L}}_{S}=1,455,\\ \text{UC}{\text{L}}_{\bar{X}}=10.98,\;\;\text{LC}{\text{L}}_{\bar{X}}=0.55,\\ \text{UC}{\text{L}}_{TS}=0.732,\;\;\text{LC}{\text{L}}_{TS}=0.121,\\ \text{UC}{\text{L}}_{T\bar{X}}=1.77,\;\;\text{LC}{\text{L}}_{T\bar{X}}=1.08,\\ \text{UC}{\text{L}}_{\text{EWMA-}S}=7.495,\;\;\text{LC}{\text{L}}_{\text{EWMA-}S}=2.485. \end{array}$$

**Figure 2:**
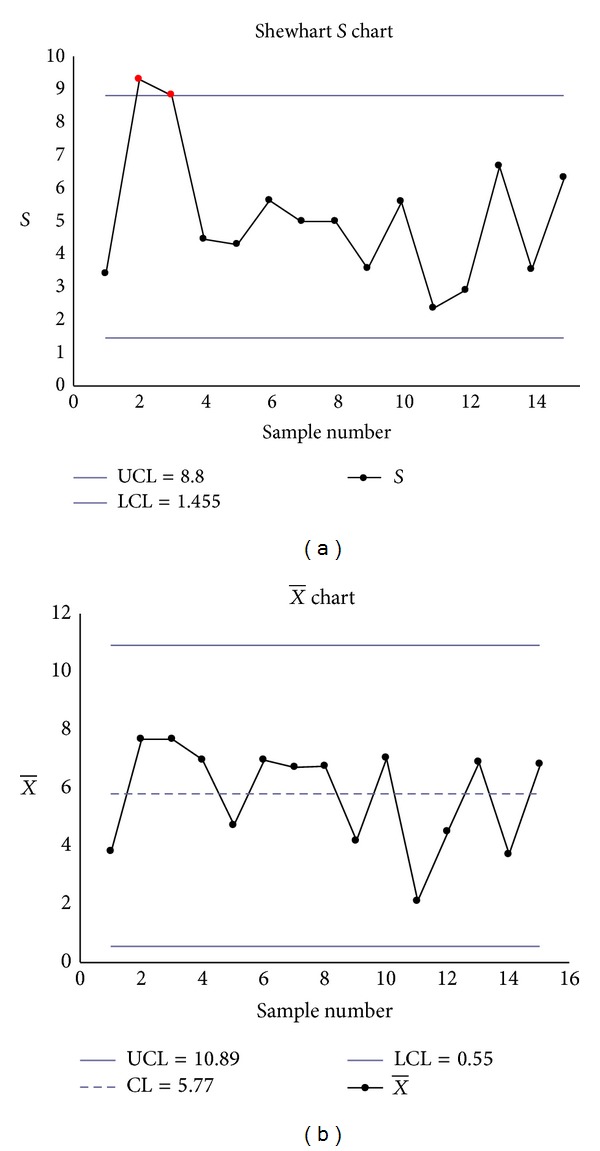
(a) Shewhart *S* chart. (b) Shewhart $\bar{X}$ chart.

**Figure 3:**
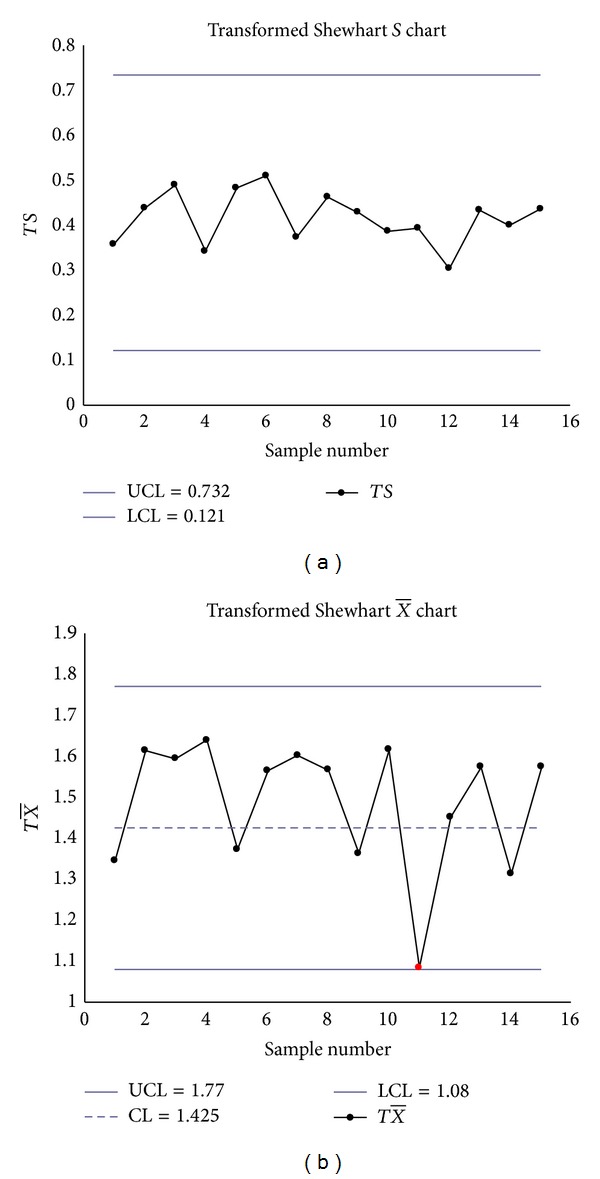
(a) The transformed Shewhart *S* chart. (b) The transformed $\bar{X}$ chart.

**Figure 4:**
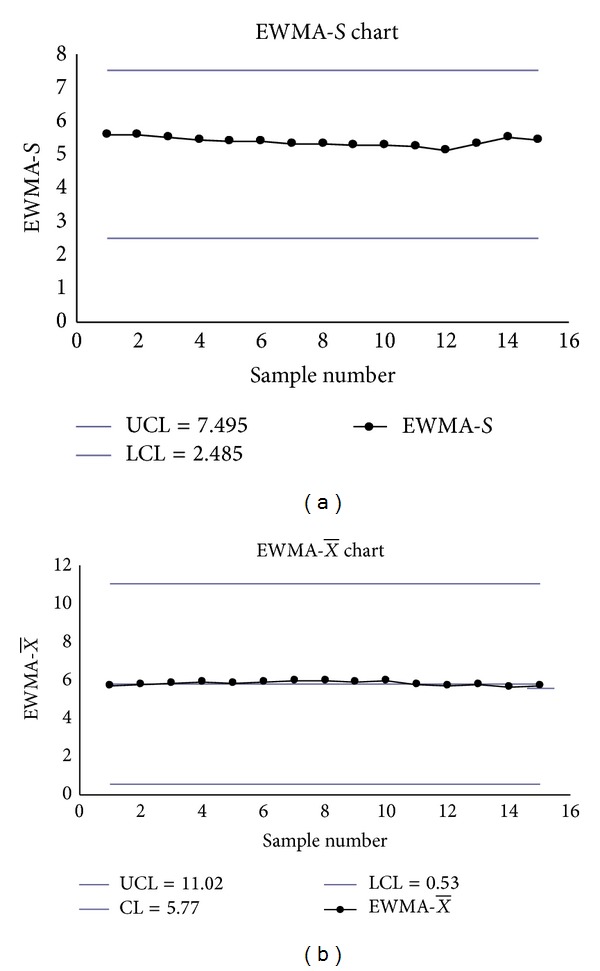
(a) The EWMA_*S*_ chart. (b) The $\text{EWM}{\text{A}}_{\bar{X}}$ chart.

The Shewhart $S\text{-}\bar{X}$ charts had two false signals (Samples 2 and 3 on the *S* chart) (see Figures 2(a) and 2(b)), the transformed $S\text{-}\bar{X}$ charts had one signal (Sample 11 on the transformed $\bar{X}$ chart) (see Figures 3(a) and 3(b)), but the EWMA-*S* and EWMA-$\bar{X}$ charts had no signals (see Figures 4(a) and 4(b)).

To illustrate the out-of-control detection ability of the service times from the new proposed EWMA-AV and EWMA-AM charts for the new automatic service system of the bank branch, 10 new samples of size 20 and new samples 1–10 were collected and listed in Table 13(b) (the last 10 observations in each new sample) and Table 13(a) (the first 10 observations in each new sample).

**Figure 5:**
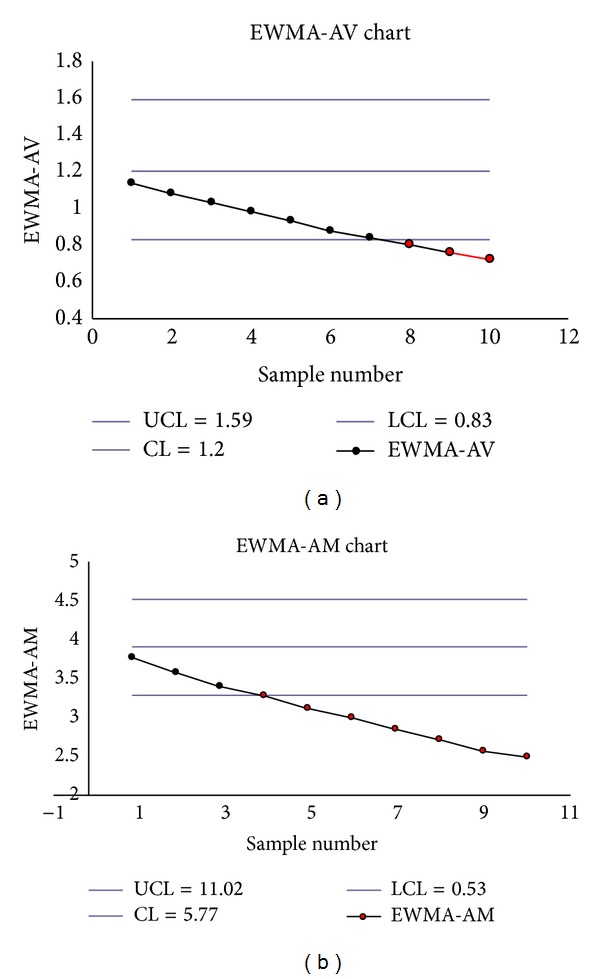
(a) The EWMA-AV chart. (b) The EWMA-AM chart.

**Figure 6:**
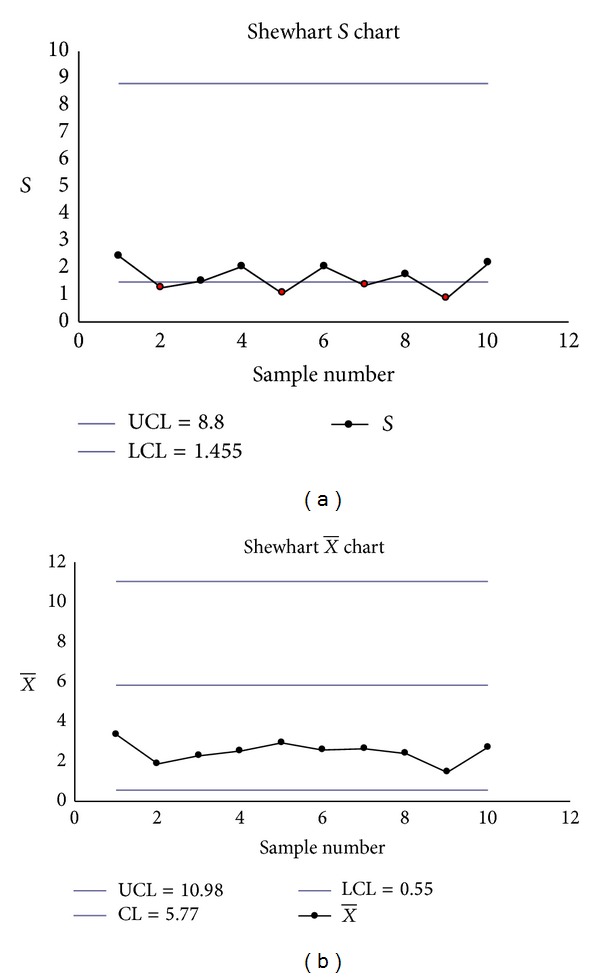
(a) The Shewhart *S* chart. (b) The Shewhart $\bar{X}$ chart.

**Figure 7:**
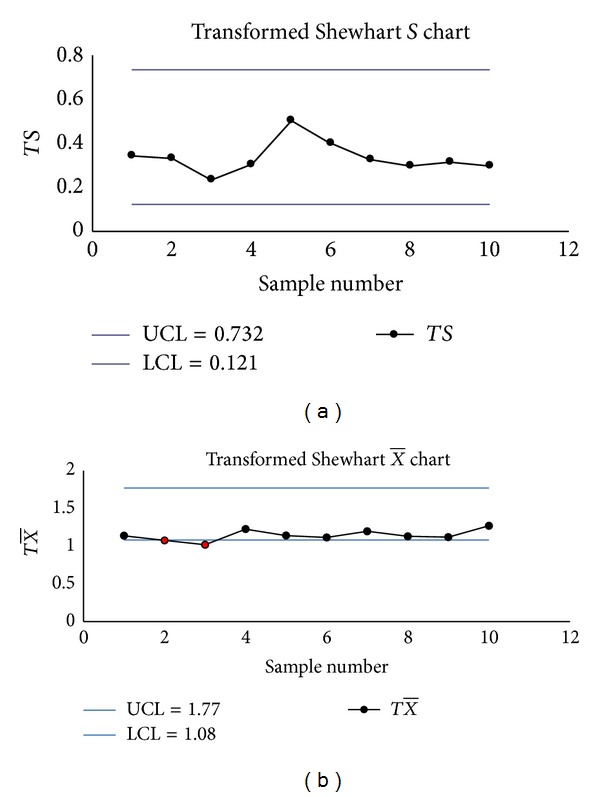
(a) The transformed Shewhart *S* chart. (b) The transformed Shewhart $\bar{X}$ chart.

**Figure 8:**
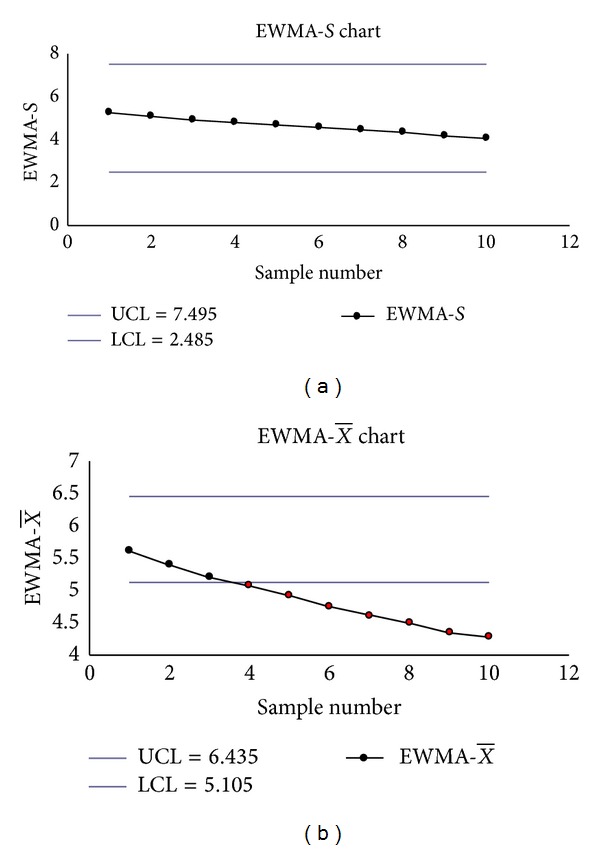
(a) The EWMA_*S*_ chart. (b) The $\text{EWM}{\text{A}}_{\bar{X}}$ chart.

Both the corresponding EWMA-AV and EWMA-AM charts, respectively, detected out-of-control variance signals from the eighth sample onward and the out-of-control mean signal from the fourth sample onward (samples 8–10 on EWMA-AV chart and samples 4–10 EWMA-AM chart) (see Figures 5(a) and 5(b)). That is, the variance and mean of the new service times are significantly reduced because of the improved new automatic service system. However, the corresponding Shewhart $S\text{-}\bar{X}$ charts produced only four true out-of-control standard deviation signals (samples 2, 5, 7, and 9 on *S* chart) (see Figures 6(a) and 6(b)), the transformed Shewhart $S\text{-}\bar{X}$ charts produced only two out-of-control mean signals (samples 2 and 3 on transformed $\bar{X}$ chart) (see Figures 7(a) and 7(b)). Both the corresponding EWMA-*S* and EWMA-$\bar{X}$ charts detected only out-of-control mean signals from the fourth sample onward (samples 4–10 on EWMA-$\bar{X}$ chart) (see Figures 8(a) and 8(b)).

Construction of the Shewhart $S\text{-}\bar{X}$ charts, the transformed $S\text{-}\bar{X}$ charts, and the EWMA-*S* and EWMA-$\bar{X}$ charts requires a normality assumption but this is not the case for the proposed EWMA-AV and EWMA-AM charts. In this example, neither the $S\text{-}\bar{X}$ charts nor the transformed $S\text{-}\bar{X}$ charts detected most of the out-of-control signals. The new EWMA-AV and EWMA-AM charts showed superior detection ability than the existing charts in monitoring and detecting process variance and mean shifts. As a consequence, in the final analysis, the EWMA-AV and EWMA-AM charts appear to be the best.

Alternatively, if we take *λ*_1_ = *λ*_2_ = 0.2 then the EWMA-AV and EWMA-AM charts are as follows based on the fifteen in-control samples in Tables 12(b) and 12(a).

 The EWMA-AV chart:
  (20) $$\begin{array}{c} \text{UC}{\text{L}}_{\text{EWMA-}V}=1.93,\;\;\text{LC}{\text{L}}_{\text{EWMA-}V}=0.47. \end{array}$$
 The EWMA-AM chart:
  (21) $$\begin{array}{c} \text{UC}{\text{L}}_{\text{EWMA-}M}=5.39,\;\;\text{LC}{\text{L}}_{\text{EWMA-}M}=2.41. \end{array}$$

**Figure 9:**
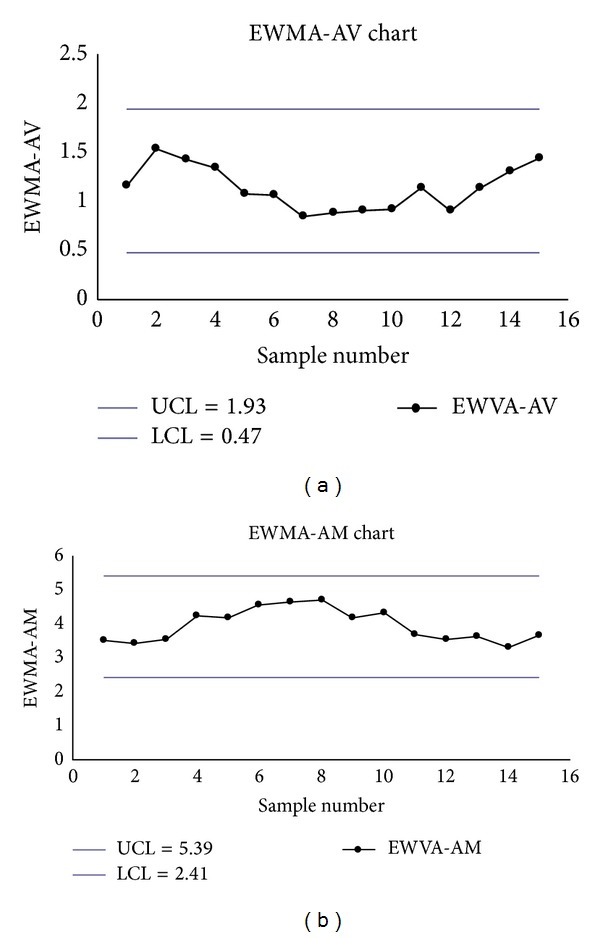
(a) The EWMA-AV chart with *λ*_2_ = 0.2. (b) The EWMA-AM chart with *λ*_1_ = 0.2.

The monitoring in-control statistics EWMA_*M*_ and EWMA_*V*_ are listed in Tables 14(b) and 14(a). The EWMA-AV and EWMA-AM charts show no signals (see Figures 9(a) and 9(b)).

The monitoring statistics of the EWMA-AM and EWMA-AV charts for the new automatic service system of the bank branch were listed in Tables 15(a) and 15(b).

**Figure 10:**
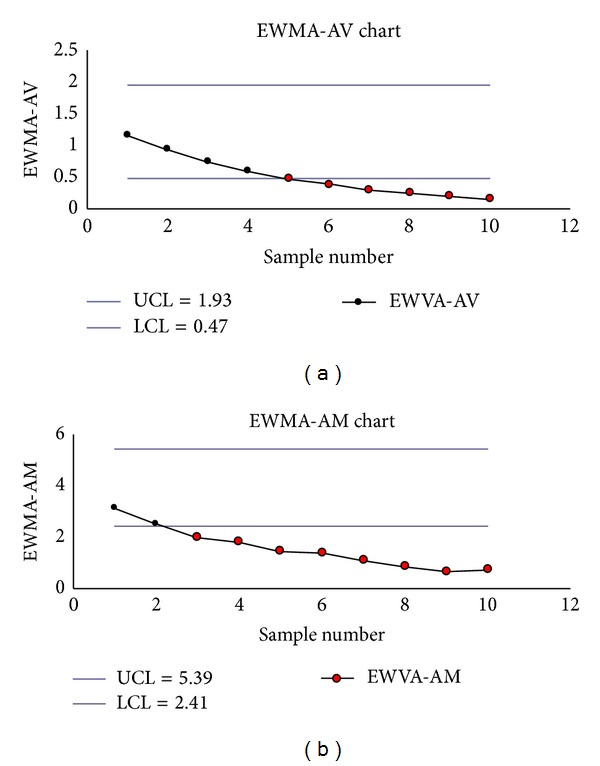
(a) The EWMA-AV chart with *λ*_2_ = 0.2. (b) The EWMA-AM chart with *λ*_1_ = 0.2.

Both the corresponding EWMA-AV and EWMA-AM charts, respectively, detected out-of-control variance signals from the fifth sample onward and the out-of-control mean signal from the third sample onward (samples 5–10 on EWMA-AV chart and samples 3–10 EWMA-AM charts) (see Figures 10(a) and 10(b)). That is, the EWMA-AV and EWMA-AM charts with *λ*_1_ = *λ*_2_ = 0.2 detected the out-of-control variance and mean earlier than that of the EWMA-AV and EWMA-AM charts with *λ*_1_ = *λ*_2_ = 0.05. The reason is that the mean and variance had larger shifts; that is, the ${\hat{p}}_{mo}=0.39$ changed to ${\hat{p}}_{m1}=0.04$ and ${\hat{p}}_{vo}=0.24$ changed to ${\hat{p}}_{v1}=0.00$. A good rule of thumb for using EWMA control chart is to use a larger weight, *λ*, to detect larger shift (see [20]).

## 7. Conclusions

In this paper, we propose using both the new EWMA-AM and EWMA-AV charts, based on two simple independent statistics to monitor the variance and mean shifts in the process simultaneously when the distribution of a quality characteristic is not known or is not believed to be normal. The EWMA-AM and EWMA-AV charts improve the detection ability of the symmetric mean chart and symmetric variance chart constructed using binomial distributions. Furthermore, the new EWMA-AM and EWMA-AV charts provide more intuitive and reasonable in-control and out-of-control average run lengths. A numerical example of service times from a bank branch with a right skewed distribution illustrated the application of the new EWMA-AM and EWMA-AV charts which were compared with some existing charts. The new EWMA-AM and EWMA-AV charts showed superior detection ability than the existing charts in monitoring and detecting both the process mean and variance shifts. The new EWMA-AM and EWMA-AV charts are thus recommended.

## Figures and Tables

**Table 1:** The *k*_3_ and *k*_4_ with ARL_*m*0_ ≈ 370 for various *n*_1_ and *p*_*m*0_ given λ_1_= 0.05.

*n*_1_	*p*_*m*0_																	
	0.10		0.20		0.30		0.40		0.5		0.6		0.7		0.8		0.9	
10	*k*_3_	2.61	*k*_3_	2.54	*k*_3_	2.49	*k*_3_	2.46	*k*_3_	2.44	*k*_3_	2.40	*k*_3_	2.31	*k*_3_	2.29	*k*_3_	2.19
	*k*_4_	2.36	*k*_4_	2.44	*k*_4_	2.49	*k*_4_	2.53	*k*_4_	2.54	*k*_4_	2.59	*k*_4_	2.72	*k*_4_	2.73	*k*_4_	2.89

11	*k*_3_	2.61	*k*_3_	2.54	*k*_3_	2.49	*k*_3_	2.44	*k*_3_	2.45	*k*_3_	2.44	*k*_3_	2.39	*k*_3_	2.32	*k*_3_	2.10
	*k*_4_	2.36	*k*_4_	2.44	*k*_4_	2.48	*k*_4_	2.54	*k*_4_	2.53	*k*_4_	2.54	*k*_4_	2.59	*k*_4_	2.67	*k*_4_	3.17

12	*k*_3_	2.60	*k*_3_	2.53	*k*_3_	2.49	*k*_3_	2.45	*k*_3_	2.45	*k*_3_	2.42	*k*_3_	2.40	*k*_3_	2.33	*k*_3_	2.35
	*k*_4_	2.36	*k*_4_	2.44	*k*_4_	2.48	*k*_4_	2.53	*k*_4_	2.54	*k*_4_	2.57	*k*_4_	2.57	*k*_4_	2.65	*k*_4_	2.62

13	*k*_3_	2.58	*k*_3_	2.53	*k*_3_	2.48	*k*_3_	2.45	*k*_3_	2.44	*k*_3_	2.40	*k*_3_	2.37	*k*_3_	2.36	*k*_3_	2.34
	*k*_4_	2.38	*k*_4_	2.43	*k*_4_	2.50	*k*_4_	2.53	*k*_4_	2.54	*k*_4_	2.58	*k*_4_	2.61	*k*_4_	2.62	*k*_4_	2.63

14	*k*_3_	2.58	*k*_3_	2.51	*k*_3_	2.49	*k*_3_	2.47	*k*_3_	2.45	*k*_3_	2.41	*k*_3_	2.38	*k*_3_	2.37	*k*_3_	2.35
	*k*_4_	2.35	*k*_4_	2.47	*k*_4_	2.49	*k*_4_	2.50	*k*_4_	2.53	*k*_4_	2.57	*k*_4_	2.60	*k*_4_	2.61	*k*_4_	2.60

15	*k*_3_	2.57	*k*_3_	2.51	*k*_3_	2.49	*k*_3_	2.47	*k*_3_	2.41	*k*_3_	2.39	*k*_3_	2.36	*k*_3_	2.43	*k*_3_	2.41
	*k*_4_	2.37	*k*_4_	2.47	*k*_4_	2.49	*k*_4_	2.51	*k*_4_	2.58	*k*_4_	2.60	*k*_4_	2.64	*k*_4_	2.55	*k*_4_	2.53

16	*k*_3_	2.57	*k*_3_	2.51	*k*_3_	2.46	*k*_3_	2.47	*k*_3_	2.44	*k*_3_	2.43	*k*_3_	2.39	*k*_3_	2.37	*k*_3_	2.45
	*k*_4_	2.38	*k*_4_	2.46	*k*_4_	2.52	*k*_4_	2.50	*k*_4_	2.54	*k*_4_	2.55	*k*_4_	2.60	*k*_4_	2.62	*k*_4_	2.51

17	*k*_3_	2.57	*k*_3_	2.51	*k*_3_	2.48	*k*_3_	2.42	*k*_3_	2.44	*k*_3_	2.40	*k*_3_	2.39	*k*_3_	2.06	*k*_3_	2.44
	*k*_4_	2.41	*k*_4_	2.46	*k*_4_	2.50	*k*_4_	2.56	*k*_4_	2.55	*k*_4_	2.59	*k*_4_	2.59	*k*_4_	2.63	*k*_4_	2.53

18	*k*_3_	2.57	*k*_3_	2.51	*k*_3_	2.47	*k*_3_	2.44	*k*_3_	2.44	*k*_3_	2.40	*k*_3_	2.37	*k*_3_	2.37	*k*_3_	2.39
	*k*_4_	2.40	*k*_4_	2.46	*k*_4_	2.51	*k*_4_	2.54	*k*_4_	2.54	*k*_4_	2.59	*k*_4_	2.62	*k*_4_	2.61	*k*_4_	2.59

19	*k*_3_	2.57	*k*_3_	2.51	*k*_3_	2.48	*k*_3_	2.47	*k*_3_	2.47	*k*_3_	2.43	*k*_3_	2.27	*k*_3_	2.20	*k*_3_	2.46
	*k*_4_	2.41	*k*_4_	2.47	*k*_4_	2.50	*k*_4_	2.51	*k*_4_	2.51	*k*_4_	2.55	*k*_4_	2.79	*k*_4_	2.94	*k*_4_	2.51

20	*k*_3_	2.55	*k*_3_	2.51	*k*_3_	2.46	*k*_3_	2.47	*k*_3_	2.44	*k*_3_	2.42	*k*_3_	2.42	*k*_3_	2.42	*k*_3_	2.5
	*k*_4_	2.42	*k*_4_	2.46	*k*_4_	2.51	*k*_4_	2.50	*k*_4_	2.55	*k*_4_	2.55	*k*_4_	2.56	*k*_4_	2.56	*k*_4_	2.48

21	*k*_3_	2.55	*k*_3_	2.49	*k*_3_	2.47	*k*_3_	2.46	*k*_3_	2.45	*k*_3_	2.42	*k*_3_	2.50	*k*_3_	2.40	*k*_3_	2.5
	*k*_4_	2.42	*k*_4_	2.48	*k*_4_	2.51	*k*_4_	2.52	*k*_4_	2.54	*k*_4_	2.56	*k*_4_	2.48	*k*_4_	2.57	*k*_4_	2.48

22	*k*_3_	2.55	*k*_3_	2.51	*k*_3_	2.48	*k*_3_	2.43	*k*_3_	2.42	*k*_3_	2.36	*k*_3_	2.29	*k*_3_	2.42	*k*_3_	2.47
	*k*_4_	2.42	*k*_4_	2.47	*k*_4_	2.51	*k*_4_	2.55	*k*_4_	2.57	*k*_4_	2.64	*k*_4_	2.76	*k*_4_	2.57	*k*_4_	2.5

23	*k*_3_	2.56	*k*_3_	2.50	*k*_3_	2.49	*k*_3_	2.46	*k*_3_	2.45	*k*_3_	2.47	*k*_3_	2.32	*k*_3_	2.47	*k*_3_	2.6
	*k*_4_	2.41	*k*_4_	2.48	*k*_4_	2.47	*k*_4_	2.52	*k*_4_	2.53	*k*_4_	2.50	*k*_4_	2.71	*k*_4_	2.51	*k*_4_	2.4

24	*k*_3_	2.54	*k*_3_	2.50	*k*_3_	2.47	*k*_3_	2.45	*k*_3_	2.44	*k*_3_	2.34	*k*_3_	2.43	*k*_3_	2.43	*k*_3_	2.52
	*k*_4_	2.42	*k*_4_	2.48	*k*_4_	2.50	*k*_4_	2.53	*k*_4_	2.54	*k*_4_	2.69	*k*_4_	2.55	*k*_4_	2.55	*k*_4_	2.45

25	*k*_3_	2.56	*k*_3_	2.50	*k*_3_	2.48	*k*_3_	2.45	*k*_3_	2.46	*k*_3_	2.35	*k*_3_	2.43	*k*_3_	2.37	*k*_3_	2.28
	*k*_4_	2.41	*k*_4_	2.48	*k*_4_	2.50	*k*_4_	2.52	*k*_4_	2.53	*k*_4_	2.68	*k*_4_	2.56	*k*_4_	2.62	*k*_4_	2.74

26	*k*_3_	2.56	*k*_3_	2.49	*k*_3_	2.47	*k*_3_	2.45	*k*_3_	2.45	*k*_3_	2.42	*k*_3_	2.44	*k*_3_	2.43	*k*_3_	2.43
	*k*_4_	2.41	*k*_4_	2.49	*k*_4_	2.50	*k*_4_	2.54	*k*_4_	2.53	*k*_4_	2.56	*k*_4_	2.55	*k*_4_	2.54	*k*_4_	2.53

27	*k*_3_	2.54	*k*_3_	2.50	*k*_3_	2.47	*k*_3_	2.46	*k*_3_	2.49	*k*_3_	2.45	*k*_3_	2.42	*k*_3_	2.40	*k*_3_	2.37
	*k*_4_	2.42	*k*_4_	2.48	*k*_4_	2.51	*k*_4_	2.52	*k*_4_	2.49	*k*_4_	2.53	*k*_4_	2.56	*k*_4_	2.58	*k*_4_	2.60

28	*k*_3_	2.55	*k*_3_	2.49	*k*_3_	2.47	*k*_3_	2.46	*k*_3_	2.35	*k*_3_	2.40	*k*_3_	2.40	*k*_3_	2.35	*k*_3_	2.30
	*k*_4_	2.42	*k*_4_	2.49	*k*_4_	2.51	*k*_4_	2.52	*k*_4_	2.67	*k*_4_	2.59	*k*_4_	2.58	*k*_4_	2.66	*k*_4_	2.71

29	*k*_3_	2.54	*k*_3_	2.50	*k*_3_	2.48	*k*_3_	2.44	*k*_3_	2.46	*k*_3_	2.41	*k*_3_	2.45	*k*_3_	2.44	*k*_3_	2.28
	*k*_4_	2.43	*k*_4_	2.48	*k*_4_	2.51	*k*_4_	2.55	*k*_4_	2.52	*k*_4_	2.57	*k*_4_	2.53	*k*_4_	2.54	*k*_4_	2.75

30	*k*_3_	2.53	*k*_3_	2.50	*k*_3_	2.47	*k*_3_	2.47	*k*_3_	2.37	*k*_3_	2.43	*k*_3_	2.42	*k*_3_	2.42	*k*_3_	2.17
	*k*_4_	2.44	*k*_4_	2.47	*k*_4_	2.51	*k*_4_	2.51	*k*_4_	2.63	*k*_4_	2.55	*k*_4_	2.56	*k*_4_	2.56	*k*_4_	3.02

**Table 2:** The ARL_*m*1_ of the EWMA-AM chart (λ_1_ = 0.05, *p*_*m*0_ = 0.1 with ARL_*m*0_ ≈ 370).

*n*_1_	*p*_*m*1_								
	0.1	0.2	0.3	0.4	0.5	0.6	0.7	0.8	0.9
10	370.3	10.4	4.9	3.3	2.5	2.1	1.9	1.7	1.3
11	372.0	9.8	4.6	3.1	2.5	2.1	1.9	1.7	1.3
12	370.4	9.3	4.4	3.0	2.3	2.0	1.8	1.4	1.1
13	370.1	8.9	4.2	2.9	2.2	1.9	1.6	1.3	1.0
14	371.9	8.5	4.1	2.7	2.2	1.9	1.6	1.3	1.0
15	371.3	8.2	4.0	2.7	2.1	1.8	1.5	1.2	1.0
16	370.3	7.9	3.8	2.6	2.1	1.8	1.6	1.2	1.0
17	369.4	7.7	3.7	2.6	2.0	1.7	1.4	1.1	1.0
18	371.0	7.4	3.7	2.5	2.0	1.6	1.3	1.1	1.0
19	368.8	7.2	3.5	2.4	2.0	1.7	1.3	1.1	1.0
20	368.8	7.0	3.5	2.4	1.9	1.6	1.2	1.0	1.0
21	370.2	6.8	3.4	2.4	1.9	1.7	1.3	1.0	1.0
22	369.9	6.6	3.3	2.3	1.9	1.5	1.2	1.0	1.0
23	370.2	6.5	3.2	2.3	1.8	1.4	1.1	1.0	1.0
24	369.9	6.3	3.1	2.2	1.9	1.5	1.2	1.0	1.0
25	370.1	6.2	3.1	2.2	1.8	1.4	1.1	1.0	1.0
26	369.8	6.0	3.0	2.1	1.7	1.3	1.1	1.0	1.0
27	369.8	5.9	3.0	2.2	1.8	1.4	1.1	1.0	1.0
28	369.7	5.8	2.9	2.1	1.7	1.3	1.0	1.0	1.0
29	369.4	5.7	2.9	2.1	1.6	1.2	1.0	1.0	1.0
30	369.9	5.6	2.9	2.1	1.7	1.3	1.0	1.0	1.0

**Table 3:** The ARL_*m*1_ of the EWMA-AM chart (λ_1_ = 0.05, *p*_*m*0_ = 0.3 with ARL_*m*0_ ≈ 370).

*n*_1_	*p*_*m*1_								
	0.1	0.2	0.3	0.4	0.5	0.6	0.7	0.8	0.9
10	7.3	17.1	373.7	17.0	7.4	4.8	3.7	2.9	2.3
11	6.8	16.0	370.0	16.0	7.0	4.6	3.4	2.8	2.3
12	6.5	15.1	370.1	15.1	6.7	4.4	3.4	2.8	2.2
13	6.3	14.4	369.8	14.4	6.4	4.2	3.2	2.6	2.1
14	6.1	13.7	370.4	13.8	6.2	4.1	3.1	2.5	2.1
15	5.9	13.1	369.9	13.2	6.0	4.0	3.0	2.4	2.0
16	5.7	12.7	369.8	12.6	5.7	3.8	2.9	2.3	2.0
17	5.5	12.2	369.8	12.2	5.6	3.7	2.8	2.2	2.0
18	5.4	11.7	370.1	11.8	5.4	3.6	2.7	2.2	2.0
19	5.2	11.3	370.2	11.4	5.2	3.5	2.7	2.1	2.0
20	5.0	11.0	370.1	11.0	5.1	3.4	2.6	2.1	2.0
21	4.9	10.7	370.1	10.7	5.0	3.3	2.5	2.1	2.0
22	4.8	10.4	370.0	10.4	4.9	3.2	2.5	2.0	2.0
23	4.7	10.1	369.9	10.2	4.8	3.2	2.4	2.0	2.0
24	4.6	9.9	369.7	9.9	4.7	3.1	2.4	2.0	2.0
25	4.5	9.6	370.0	9.7	4.6	3.1	2.3	2.0	2.0
26	4.4	9.4	370.0	9.5	4.5	3.0	2.3	2.0	2.0
27	4.3	9.2	370.5	9.3	4.4	3.0	2.2	2.0	2.0
28	4.3	9.0	370.0	9.1	4.3	2.9	2.2	2.0	1.9
29	4.2	8.8	370.1	8.9	4.2	2.9	2.2	2.0	2.0
30	4.1	8.7	369.8	8.7	4.2	2.8	2.2	2.0	2.0

**Table 4:** The ARL_*m*1_ of the EWMA-AM chart (λ_1_ = 0.05, *p*_*m*0_ = 0.5 with ARL_*m*0_ ≈ 370).

*n*_1_	*p*_*m*1_								
	0.1	0.2	0.3	0.4	0.5	0.6	0.7	0.8	0.9
10	3.9	5.2	8.2	19.4	369.5	18.8	8.1	5.2	3.9
11	3.7	5.0	7.7	18.2	369.9	17.7	7.6	5.0	3.7
12	3.5	4.8	7.4	17.2	369.7	16.7	7.3	4.7	3.5
13	3.4	4.6	7.1	16.3	369.6	15.8	7.0	4.5	3.4
14	3.2	4.4	6.8	15.5	370.3	15.2	6.7	4.4	3.2
15	3.2	4.2	6.5	15.1	370.0	14.4	6.4	4.2	3.2
16	3.1	4.1	6.3	14.3	370.2	13.9	6.2	4.1	3.1
17	3.1	4.0	6.1	13.7	370.0	13.4	6.0	3.9	3.0
18	3.0	3.9	5.9	13.3	370.0	12.9	5.9	3.8	3.0
19	2.9	3.7	5.7	12.7	370.0	12.6	5.7	3.7	2.9
20	2.9	3.7	5.6	12.4	370.0	12.1	5.5	3.7	2.9
21	2.8	3.6	5.4	12.0	369.9	11.8	5.4	3.6	2.8
22	2.8	3.5	5.3	11.8	369.5	11.4	5.3	3.5	2.8
23	2.7	3.4	5.2	11.4	369.9	11.2	5.2	3.4	2.7
24	2.7	3.4	5.1	11.1	370.0	10.9	5.0	3.4	2.7
25	2.6	3.3	5.0	10.8	370.0	10.7	4.9	3.3	2.6
26	2.4	3.3	4.9	10.5	370.3	10.4	4.8	3.3	2.4
27	2.5	3.2	4.8	10.2	369.3	10.2	4.8	3.2	2.5
28	2.3	3.2	4.8	10.4	369.9	9.8	4.7	3.2	2.3
29	2.3	3.1	4.6	9.9	370.0	9.7	4.6	3.1	2.2
30	2.2	3.1	4.6	9.9	369.8	9.5	4.5	3.1	2.2

**Table 5:** The ARL_1_ of the symmetric EWMA mean chart (*λ*_1_ = 0.05, *p*_*m*0_ = 0.5 with ARL_*m*0_ ≈ 370).

*n*_1_	*p*_*m*1_								
	0.1	0.2	0.3	0.4	0.5	0.6	0.7	0.8	0.9
10	4	5	8	19	371	19	8	5	4
11	4	5	8	18	370	18	8	5	4
12	4	5	7	17	380	17	7	5	4
13	3	5	7	16	377	16	7	5	3
14	3	4	7	15	378	15	7	4	3
15	3	4	7	15	386	15	7	4	3
16	3	4	6	14	371	14	6	4	3
17	3	4	6	14	384	14	6	4	3
18	3	4	6	13	375	13	6	4	3
19	3	4	6	13	388	13	6	4	3
20	3	4	6	12	389	12	6	4	3
21	3	4	5	12	379	12	5	4	3
22	3	4	5	12	383	12	5	4	3
23	3	4	5	11	383	11	5	3	3
24	3	3	5	11	381	11	5	3	3
25	3	3	5	11	377	11	5	3	3

**Table 6:** The *L*_3_ and *L*_4_ with ARL_*v*0_ ≈ 370 for various 0.5*n*_2_ and *p*_*v*0_ given λ_2_ = 0.05.

0.5*n*_2_	*p*_*v*0_							
	0.10		0.20		0.30		0.40	
5	*L*_3_	2.69	*L*_3_	2.58	*L*_3_	2.51	*L*_3_	2.49
	*L*_4_	2.26	*L*_4_	2.39	*L*_4_	2.45	*L*_4_	2.48

6	*L*_3_	2.66	*L*_3_	2.58	*L*_3_	2.51	*L*_3_	2.48
	*L*_4_	2.28	*L*_4_	2.39	*L*_4_	2.46	*L*_4_	2.48

7	*L*_3_	2.62	*L*_3_	2.55	*L*_3_	2.52	*L*_3_	2.47
	*L*_4_	2.31	*L*_4_	2.39	*L*_4_	2.46	*L*_4_	2.52

8	*L*_3_	2.63	*L*_3_	2.54	*L*_3_	2.49	*L*_3_	2.47
	*L*_4_	2.32	*L*_4_	2.42	*L*_4_	2.48	*L*_4_	2.51

9	*L*_3_	2.62	*L*_3_	2.54	*L*_3_	2.52	*L*_3_	2.45
	*L*_4_	2.34	*L*_4_	2.43	*L*_4_	2.45	*L*_4_	2.53

10	*L*_3_	2.61	*L*_3_	2.54	*L*_3_	2.49	*L*_3_	2.46
	*L*_4_	2.36	*L*_4_	2.44	*L*_4_	2.49	*L*_4_	2.53

11	*L*_3_	2.61	*L*_3_	2.54	*L*_3_	2.49	*L*_3_	2.44
	*L*_4_	2.36	*L*_4_	2.44	*L*_4_	2.48	*L*_4_	2.54

12	*L*_3_	2.60	*L*_3_	2.53	*L*_3_	2.49	*L*_3_	2.45
	*L*_4_	2.36	*L*_4_	2.44	*L*_4_	2.48	*L*_4_	2.53

13	*L*_3_	2.58	*L*_3_	2.53	*L*_3_	2.48	*L*_3_	2.45
	*L*_4_	2.38	*L*_4_	2.43	*L*_4_	2.50	*L*_4_	2.53

14	*L*_3_	2.58	*L*_3_	2.51	*L*_3_	2.49	*L*_3_	2.47
	*L*_4_	2.35	*L*_4_	2.47	*L*_4_	2.49	*L*_4_	2.50

15	*L*_3_	2.57	*L*_3_	2.51	*L*_3_	2.49	*L*_3_	2.47
	*L*_4_	2.37	*L*_4_	2.47	*L*_4_	2.49	*L*_4_	2.51

**Table 7:** The ARL_*v*1_ of the EWMA-AV chart for λ_2_ = 0.05 and *p*_*v*0_ = 0.1.

0.5*n*_2_	*p*_*v*1_								
	0.1	0.2	0.3	0.4	0.5	0.6	0.7	0.8	0.9
5	375.3	16.1	7.1	4.7	3.5	2.9	2.4	2.1	2.0
6	374.2	14.3	6.4	4.2	3.2	2.7	2.3	2.1	2.0
7	370.1	13.0	5.9	3.8	2.8	2.3	2.0	1.9	1.6
8	369.9	12.0	5.5	3.6	2.8	2.3	2.0	1.9	1.6
9	368.8	11.1	5.2	3.5	2.7	2.3	2.0	1.9	1.6
10	370.3	10.4	4.9	3.3	2.5	2.1	1.9	1.7	1.3
11	372.0	9.8	4.6	3.1	2.5	2.1	1.9	1.7	1.3
12	370.4	9.3	4.4	3.0	2.3	2.0	1.8	1.4	1.1
13	370.1	8.9	4.2	2.9	2.2	1.9	1.6	1.3	1.0
14	371.9	8.5	4.1	2.7	2.2	1.9	1.6	1.3	1.0
15	371.3	8.2	4.0	2.7	2.1	1.8	1.5	1.2	1.0

**Table 8:** The ARL_*v*1_ of the EWMA-AV chart for λ_2_ = 0.05 and *p*_*v*0_ = 0.4.

0.5*n*_2_	*p*_*v*1_								
	0.1	0.2	0.3	0.4	0.5	0.6	0.7	0.8	0.9
5	7.2	11.8	30.2	370.1	29.8	11.9	7.4	5.4	4.4
6	6.6	10.6	26.6	370.5	26.2	10.7	6.7	4.9	4.0
7	6.1	9.7	24.0	370.0	23.3	9.7	6.2	4.6	3.7
8	5.8	9.0	21.8	369.6	21.4	9.0	5.7	4.3	3.4
9	5.4	8.4	20.4	370.1	19.6	8.4	5.4	4.0	3.3
10	5.1	7.9	18.9	369.9	18.4	7.9	5.1	3.8	3.1
11	4.9	7.5	17.7	369.9	17.2	7.5	4.9	3.6	3.0
12	4.6	7.2	16.7	370.0	16.3	7.1	4.6	3.5	2.9
13	4.5	6.8	15.8	369.7	15.5	6.9	4.5	3.4	2.8
14	4.3	6.6	15.0	370.1	14.9	6.6	4.3	3.3	2.8
15	4.1	6.3	14.3	370.1	14.3	6.4	4.2	3.2	2.6

**Table 9:** The ARL_1_ of the EWMA-AM and EWMA-AV charts for λ_1_ = λ_2_ = 0.05 and *p*_*m*0_ = *p*_*v*0_ = 0.1.

*n*_1_	0.5*n*_2_	*p*_*v*1_ = 0.1 *p*_*m*1_			*p*_*v*1_ = 0.3 *p*_*m*1_			*p*_*v*1_ = 0.7 *p*_*m*1_		
		0.1	0.3	0.7	0.1	0.3	0.7	0.1	0.3	0.7
10	5	186.4	4.8	1.9	7.0	2.9	1.5	2.4	1.6	1.1
12	6	186.1	4.3	1.8	6.3	2.6	1.4	2.3	1.5	1.0
14	7	185.5	4.1	1.6	5.8	2.4	1.2	2.0	1.3	0.9
16	8	185.0	3.8	1.6	5.4	2.2	1.2	2.0	1.3	0.9
18	9	185.0	3.7	1.3	5.1	2.2	1.0	2.0	1.3	0.8
20	10	184.8	3.5	1.2	4.8	2.0	1.0	1.9	1.2	0.7
22	11	185.5	3.3	1.2	4.5	1.9	1.0	1.9	1.2	0.7
24	12	185.1	3.1	1.2	4.3	1.8	0.9	1.8	1.1	0.7
26	13	185.0	3.0	1.1	4.2	1.8	0.9	1.6	1.0	0.7
28	14	185.4	2.9	1.0	4.1	1.7	0.8	1.6	1.0	0.6
30	15	185.3	2.9	1.0	4.0	1.7	0.8	1.5	1.0	0.6

**Table 10:** The ARL_1_ of the EWMA-AM and EWMA-AV charts for λ_1_ = λ_2_ = 0.05, *p*_*m*0_ = 0.3, and *p*_*v*0_ = 0.1.

*n*_1_	0.5*n*_2_	*p*_*v*1_ = 0.1 *p*_*m*1_			*p*_*v*1_ = 0.3 *p*_*m*1_			*p*_*v*1_ = 0.7 *p*_*m*1_		
		0.1	0.3	0.7	0.1	0.3	0.7	0.1	0.3	0.7
10	5	7.2	187.2	3.7	3.6	7.0	2.4	1.8	2.4	1.5
12	6	6.4	186.1	3.4	3.2	6.3	2.2	1.7	2.3	1.4
14	7	6.0	185.1	3.1	3.0	5.8	2.0	1.5	2.0	1.2
16	8	5.6	185.0	2.9	2.8	5.4	1.9	1.5	2.0	1.2
18	9	5.3	184.7	2.7	2.7	5.1	1.8	1.5	2.0	1.1
20	10	4.9	185.1	2.6	2.5	4.8	1.7	1.4	1.9	1.1
22	11	4.7	185.5	2.5	2.3	4.5	1.6	1.4	1.9	1.1
24	12	4.5	185.0	2.4	2.2	4.3	1.6	1.3	1.8	1.0
26	13	4.3	185.0	2.3	2.1	4.2	1.5	1.2	1.6	0.9
28	14	4.3	185.5	2.2	2.1	4.1	1.4	1.2	1.6	0.9
30	15	4.1	185.3	2.2	2.0	4.0	1.4	1.1	1.5	0.9

**Table 11:** The ARL_1_ of the EWMA-AM and EWMA-AV charts for λ_1_ = *λ*_2_ = 0.05, *p*_*m*0_ = 0.5, and *p*_*v*0_ = 0.4.

*n*_1_	0.5*n*_2_	*p*_*v*1_ = 0.1 *p*_*m*1_			*p*_*v*1_ = 0.3 *p*_*m*1_			*p*_*v*1_ = 0.7 *p*_*m*1_		
		0.1	0.3	0.7	0.1	0.3	0.7	0.1	0.3	0.7
10	5	2.6	3.8	3.8	3.4	6.4	6.4	2.6	3.9	3.9
12	6	2.3	3.5	3.5	3.1	5.8	5.7	2.3	3.5	3.5
14	7	2.1	3.2	3.2	2.8	5.3	5.2	2.1	3.2	3.2
16	8	2.0	3.0	3.0	2.7	4.9	4.8	2.0	3.0	3.0
18	9	1.9	2.8	2.8	2.6	4.6	4.6	1.9	2.8	2.8
20	10	1.8	2.7	2.6	2.5	4.3	4.2	1.8	2.7	2.6
22	11	1.8	2.5	2.5	2.4	4.1	4.1	1.8	2.5	2.5
24	12	1.7	2.4	2.4	2.3	3.9	3.8	1.7	2.4	2.4
26	13	1.6	2.3	2.3	2.1	3.7	3.7	1.6	2.3	2.3
28	14	1.5	2.3	2.2	2.0	3.6	3.6	1.5	2.3	2.2
30	15	1.4	2.2	2.1	1.9	3.5	3.4	1.4	2.2	2.2

**Table:** (a)

*t*	*X*_1_	*X*_2_	*X*_3_	*X*_4_	*X*_5_	*X*_6_	*X*_7_	*X*_8_	*X*_9_	*X*_10_	*M*_*t*_	EWMA_*M*_*i*__
1	0.88	0.78	5.06	5.45	2.93	6.11	11.59	1.20	0.89	3.21	2	3.81
2	3.82	13.4	5.16	3.20	32.27	3.68	3.14	1.58	2.72	7.71	3	3.76
3	1.40	3.89	10.88	30.85	0.54	8.40	5.10	2.63	9.17	3.94	4	3.78
4	16.8	8.77	8.36	3.55	7.76	1.81	1.11	5.91	8.26	7.19	7	3.94
5	0.24	9.57	0.66	1.15	2.34	0.57	8.94	5.54	11.69	6.58	4	3.94
6	4.21	8.73	11.44	2.89	19.49	1.20	8.01	6.19	7.48	0.07	6	4.04
7	15.08	7.43	4.31	6.14	10.37	2.33	1.97	1.08	4.27	14.08	5	4.09
8	13.89	0.30	3.21	11.32	9.90	4.39	10.5	1.70	10.74	1.46	5	4.14
9	0.03	12.76	2.41	7.41	1.67	3.70	4.31	2.45	3.57	3.33	2	4.03
10	12.89	17.96	2.78	3.21	1.12	12.61	4.23	6.18	2.33	6.92	5	4.08
11	7.71	1.05	1.11	0.22	3.53	0.81	0.41	3.73	0.08	2.55	1	3.92
12	5.81	6.29	3.46	2.66	4.02	10.95	1.59	5.58	0.55	4.10	3	3.88
13	2.89	1.61	1.30	2.58	18.65	10.77	18.23	3.13	3.38	6.34	4	3.88
14	1.36	1.92	0.12	11.08	8.85	3.99	4.32	1.71	1.77	1.94	2	3.79
15	21.52	0.63	8.54	3.37	6.94	3.44	3.37	6.37	1.28	12.83	5	3.85

**Table:** (b)

*t*	*X*_11_	*X*_12_	*X*_13_	*X*_14_	*X*_15_	*X*_16_	*X*_17_	*X*_18_	*X*_19_	*X*_20_	*V*_*t*_	EWMA_*V*_*i*__
1	3.82	6.29	10.88	30.85	9.9	3.99	1.59	1.71	8.26	4.1	1	1.19
2	0.24	12.76	11.44	3.2	3.53	0.57	18.23	2.45	2.72	6.92	3	1.28
3	3.82	7.43	0.12	3.37	1.12	12.61	1.59	1.08	0.89	0.07	1	1.27
4	13.89	3.89	5.16	11.32	4.02	0.57	8.01	6.19	1.77	6.58	1	1.25
5	5.81	12.76	2.41	1.15	3.53	0.81	11.59	5.91	4.27	3.33	0	1.19
6	12.89	8.73	10.88	2.89	18.65	10.95	0.41	3.13	4.27	7.71	1	1.18
7	2.89	0.63	0.12	0.22	4.02	10.95	8.01	1.08	10.74	4.1	0	1.12
8	16.8	1.05	1.3	3.2	2.34	0.81	4.32	3.13	0.08	1.46	1	1.12
9	4.21	17.96	5.06	0.22	4.02	3.99	8.01	5.91	0.55	3.33	1	1.11
10	12.89	8.77	11.44	7.41	1.12	1.81	4.32	5.58	0.89	14.08	1	1.10
11	0.88	8.77	5.06	3.55	8.85	10.95	18.23	5.54	2.33	6.58	2	1.15
12	7.71	7.43	0.12	2.58	1.12	2.33	4.23	2.63	4.27	3.33	0	1.09
13	7.71	9.57	0.12	30.85	7.76	1.81	3.14	1.71	2.72	14.08	2	1.14
14	2.89	1.05	2.41	11.32	32.27	8.4	1.97	2.45	11.69	12.83	2	1.18
15	1.36	0.63	3.46	11.32	0.54	10.95	4.23	2.45	2.33	6.34	2	1.22

**Table:** (a)

*t*	*X*_1_	*X*_2_	*X*_3_	*X*_4_	*X*_5_	*X*_6_	*X*_7_	*X*_8_	*X*_9_	*X*_10_	*M*_*t*_	EWMA_*M*_*i*__
1	3.54	0.01	1.33	7.27	5.52	0.09	1.84	1.04	2.91	0.63	1	3.76
2	0.86	1.61	1.15	0.96	0.54	3.05	4.11	0.63	2.37	0.05	0	3.57
3	1.45	0.19	4.18	0.18	0.02	0.70	0.80	0.97	3.60	2.94	0	3.39
4	1.37	0.14	1.54	1.58	0.45	6.01	4.59	1.74	3.92	4.82	1	3.27
5	3.00	2.46	0.06	1.80	3.25	2.13	2.22	1.37	2.13	0.25	0	3.11
6	1.59	3.88	0.39	0.54	1.58	1.70	0.68	1.25	6.83	0.31	1	3.00
7	5.01	1.85	3.10	1.00	0.09	1.16	2.69	2.79	1.84	2.62	0	2.85
8	4.96	0.55	1.43	4.12	4.06	1.42	1.43	0.86	0.67	0.13	0	2.71
9	1.08	0.65	0.91	0.88	2.02	2.88	1.76	2.87	1.97	0.62	0	2.57
10	4.56	0.44	5.61	2.79	1.73	2.46	0.53	1.73	7.02	2.13	1	2.49

**Table:** (b)

*t*	*X*_11_	*X*_12_	*X*_13_	*X*_14_	*X*_15_	*X*_16_	*X*_17_	*X*_18_	*X*_19_	*X*_20_	*V*_*t*_	EWMA_*V*_*i*__
1	5.01	3.88	4.18	0.88	4.06	3.05	4.59	0.63	6.83	0.25	0	1.14
2	1.59	0.19	0.39	0.54	1.73	1.42	4.11	1.25	7.02	0.62	0	1.08
3	1.59	0.65	1.43	1.00	5.52	1.16	4.11	1.37	3.6	2.62	0	1.03
4	3.54	0.65	1.43	1.00	4.06	6.01	4.59	1.37	1.97	0.31	0	0.98
5	5.01	0.01	1.54	7.27	0.09	2.88	4.11	0.97	7.02	0.63	0	0.93
6	4.96	0.14	0.91	4.12	4.06	6.01	2.69	0.63	2.13	0.13	0	0.88
7	4.96	2.46	1.54	7.27	0.45	1.42	0.53	0.63	2.13	4.82	0	0.84
8	1.37	0.44	1.33	1.00	0.45	2.46	2.22	2.87	7.02	4.82	0	0.80
9	1.59	0.01	0.91	1.58	0.54	1.70	1.84	2.79	2.91	0.62	0	0.76
10	5.01	0.44	0.91	0.88	1.73	3.05	1.43	1.74	6.83	4.82	0	0.72

**Table:** (a)

*t*	*X*_1_	*X*_2_	*X*_3_	*X*_4_	*X*_5_	*X*_6_	*X*_7_	*X*_8_	*X*_9_	*X*_10_	*M*_*t*_	EWMA_*M*_*i*__
1	0.88	0.78	5.06	5.45	2.93	6.11	11.59	1.20	0.89	3.21	2	3.52
2	3.82	13.4	5.16	3.20	32.27	3.68	3.14	1.58	2.72	7.71	3	3.42
3	1.40	3.89	10.88	30.85	0.54	8.40	5.10	2.63	9.17	3.94	4	3.53
4	16.8	8.77	8.36	3.55	7.76	1.81	1.11	5.91	8.26	7.19	7	4.23
5	0.24	9.57	0.66	1.15	2.34	0.57	8.94	5.54	11.69	6.58	4	4.18
6	4.21	8.73	11.44	2.89	19.49	1.20	8.01	6.19	7.48	0.07	6	4.54
7	15.08	7.43	4.31	6.14	10.37	2.33	1.97	1.08	4.27	14.08	5	4.64
8	13.89	0.30	3.21	11.32	9.90	4.39	10.5	1.70	10.74	1.46	5	4.71
9	0.03	12.76	2.41	7.41	1.67	3.70	4.31	2.45	3.57	3.33	2	4.17
10	12.89	17.96	2.78	3.21	1.12	12.61	4.23	6.18	2.33	6.92	5	4.33
11	7.71	1.05	1.11	0.22	3.53	0.81	0.41	3.73	0.08	2.55	1	3.67
12	5.81	6.29	3.46	2.66	4.02	10.95	1.59	5.58	0.55	4.10	3	3.53
13	2.89	1.61	1.30	2.58	18.65	10.77	18.23	3.13	3.38	6.34	4	3.63
14	1.36	1.92	0.12	11.08	8.85	3.99	4.32	1.71	1.77	1.94	2	3.30
15	21.52	0.63	8.54	3.37	6.94	3.44	3.37	6.37	1.28	12.83	5	3.64

**Table:** (b)

*t*	*X*_11_	*X*_12_	*X*_13_	*X*_14_	*X*_15_	*X*_16_	*X*_17_	*X*_18_	*X*_19_	*X*_20_	*V*_*t*_	EWMA_*V*_*i*__
1	3.82	6.29	10.88	30.85	9.9	3.99	1.59	1.71	8.26	4.1	1	1.16
2	0.24	12.76	11.44	3.2	3.53	0.57	18.23	2.45	2.72	6.92	3	1.53
3	3.82	7.43	0.12	3.37	1.12	12.61	1.59	1.08	0.89	0.07	1	1.42
4	13.89	3.89	5.16	11.32	4.02	0.57	8.01	6.19	1.77	6.58	1	1.34
5	5.81	12.76	2.41	1.15	3.53	0.81	11.59	5.91	4.27	3.33	0	1.07
6	12.89	8.73	10.88	2.89	18.65	10.95	0.41	3.13	4.27	7.71	1	1.06
7	2.89	0.63	0.12	0.22	4.02	10.95	8.01	1.08	10.74	4.1	0	0.85
8	16.8	1.05	1.3	3.2	2.34	0.81	4.32	3.13	0.08	1.46	1	0.88
9	4.21	17.96	5.06	0.22	4.02	3.99	8.01	5.91	0.55	3.33	1	0.90
10	12.89	8.77	11.44	7.41	1.12	1.81	4.32	5.58	0.89	14.08	1	0.92
11	0.88	8.77	5.06	3.55	8.85	10.95	18.23	5.54	2.33	6.58	2	1.14
12	7.71	7.43	0.12	2.58	1.12	2.33	4.23	2.63	4.27	3.33	0	0.91
13	7.71	9.57	0.12	30.85	7.76	1.81	3.14	1.71	2.72	14.08	2	1.13
14	2.89	1.05	2.41	11.32	32.27	8.4	1.97	2.45	11.69	12.83	2	1.30
15	1.36	0.63	3.46	11.32	0.54	10.95	4.23	2.45	2.33	6.34	2	1.44

**Table:** (a)

*t*	*X*_1_	*X*_2_	*X*_3_	*X*_4_	*X*_5_	*X*_6_	*X*_7_	*X*_8_	*X*_9_	*X*_10_	*M*_*t*_	EWMA_*M*_*i*__
1	3.54	0.01	1.33	7.27	5.52	0.09	1.84	1.04	2.91	0.63	1	3.11
2	0.86	1.61	1.15	0.96	0.54	3.05	4.11	0.63	2.37	0.05	0	2.49
3	1.45	0.19	4.18	0.18	0.02	0.70	0.80	0.97	3.60	2.94	0	1.99
4	1.37	0.14	1.54	1.58	0.45	6.01	4.59	1.74	3.92	4.82	1	1.79
5	3.00	2.46	0.06	1.80	3.25	2.13	2.22	1.37	2.13	0.25	0	1.43
6	1.59	3.88	0.39	0.54	1.58	1.70	0.68	1.25	6.83	0.31	1	1.35
7	5.01	1.85	3.10	1.00	0.09	1.16	2.69	2.79	1.84	2.62	0	1.08
8	4.96	0.55	1.43	4.12	4.06	1.42	1.43	0.86	0.67	0.13	0	0.86
9	1.08	0.65	0.91	0.88	2.02	2.88	1.76	2.87	1.97	0.62	0	0.69
10	4.56	0.44	5.61	2.79	1.73	2.46	0.53	1.73	7.02	2.13	1	0.75

**Table:** (b)

*t*	*X*_11_	*X*_12_	*X*_13_	*X*_14_	*X*_15_	*X*_16_	*X*_17_	*X*_18_	*X*_19_	*X*_20_	*V*_*t*_	EWMA_*V*_*i*__
1	5.01	3.88	4.18	0.88	4.06	3.05	4.59	0.63	6.83	0.25	0	1.15
2	1.59	0.19	0.39	0.54	1.73	1.42	4.11	1.25	7.02	0.62	0	0.92
3	1.59	0.65	1.43	1.00	5.52	1.16	4.11	1.37	3.6	2.62	0	0.74
4	3.54	0.65	1.43	1.00	4.06	6.01	4.59	1.37	1.97	0.31	0	0.59
5	5.01	0.01	1.54	7.27	0.09	2.88	4.11	0.97	7.02	0.63	0	0.47
6	4.96	0.14	0.91	4.12	4.06	6.01	2.69	0.63	2.13	0.13	0	0.38
7	4.96	2.46	1.54	7.27	0.45	1.42	0.53	0.63	2.13	4.82	0	0.30
8	1.37	0.44	1.33	1.00	0.45	2.46	2.22	2.87	7.02	4.82	0	0.24
9	1.59	0.01	0.91	1.58	0.54	1.70	1.84	2.79	2.91	0.62	0	0.19
10	5.01	0.44	0.91	0.88	1.73	3.05	1.43	1.74	6.83	4.82	0	0.15
